# MicroRNA-21 and PDCD4 expression during in vitro oocyte maturation in pigs

**DOI:** 10.1186/s12958-016-0152-2

**Published:** 2016-04-16

**Authors:** Elane C. Wright, Benjamin J. Hale, Cai-Xia Yang, Josephat G. Njoka, Jason W. Ross

**Affiliations:** Department of Animal Science, Iowa State University, 2356 Kildee hall, Ames, IA 50011 USA

**Keywords:** miRNA, Oocyte, Pig, MIR21

## Abstract

**Background:**

MicroRNA (miRNA) are small non-coding RNA molecules critical for regulating cellular function, and are abundant in the maturing oocyte and developing embryo. MiRNA-21 (MIR21) has been shown to elicit posttranscriptional gene regulation in several tissues associated with rapid cell proliferation in addition to demonstrating anti-apoptotic features through interactions with *PDCD4* mRNA and other targets. In many tissues, MIR21 interacts and suppresses PDCD4 due to the strong complementation between MIR21 and the *PDCD4* 3′UTR.

**Methods:**

The objective of this project was to examine the relationship between MIR21 and PDCD4 expression in porcine oocytes during in vitro maturation and assess the impact of MIR21 inhibition during oocyte maturation on early embryo development. Additionally, we evaluated the effect of gonadotropins in maturation media and the presence of cumulus cells to determine their ability to contribute to MIR21 abundance in the oocyte during maturation.

**Results:**

During in vitro maturation, expression of MIR21 increased approximately 6-fold in the oocyte and 25-fold in the cumulus cell. Temporally associated with this was the reduction of PDCD4 protein abundance in MII arrested oocytes compared with GV stage oocytes, although *PDCD4* mRNA was not significantly different during this transition. Neither the presence of cumulus cells nor gonadotropins during in vitro maturation affected MIR21 abundance in those oocytes achieving MII arrest. However, inhibition of MIR21 activity during in vitro maturation using antisense MIR21 suppressed embryo development to the 4–8 cell stage following parthenogenetic activation.

**Conclusions:**

MIR21 is differentially expressed in the oocyte during meiotic maturation in the pig and inhibition of MIR21 during this process alters PDCD4 protein abundance suggesting posttranscriptional regulatory events involving MIR21 during oocyte maturation may impact subsequent embryonic development in the pig.

## Background

Germinal vesicle breakdown (GVBD) is the first physical sign that an oocyte is committed to maturation and also represents the onset of a period of transcriptional quiescence which persists until the activation of the embryonic genome. During this period, changes in mRNA and protein abundance within the oocyte can occur through interactions with the surrounding cumulus cells and/or through posttranscriptional gene regulation (PTGR) within the oocyte. MicroRNA (miRNA) represent a unique RNA class that function as potent regulators of transcription and protein abundance through PTGR [12]. MiRNA are small (18–24 nt), non-coding RNA molecules that confer PTGR through several mechanisms, such as impairing translation efficiency and affecting mRNA stability following interaction with the 3′ untranslated region (3′UTR) of target mRNA molecules [5, 6]. Numerous miRNA are expressed in the mouse oocyte and developing embryo and it has been demonstrated that the conditional knockout of DICER, an enzyme involved in miRNA processing, during oocyte development impairs the production of oocytes capable of being fertilized and developing normally [38]. Estimations predict approximately 1,000 miRNA are present in the human genome having the potential ability to impact approximately 30 % of protein coding genes [28]. Some miRNA have numerous mRNA targets [27] while others have few predicted targets [8, 34].

Utilizing miRNA microarray analysis and deep sequencing we have previously identified microRNA-21 (MIR21) as an up-regulated miRNA during porcine oocyte in vitro maturation [41]. In mice it has been reported that luteinizing hormone may increase the expression of MIR21 in mouse granulosa cells and in vivo MIR21 inhibition has a negative impact on ovulation rate [11]. MIR21 is a well characterized miRNA that has demonstrated the ability to confer PTGR oncogenic cell lines by affecting cellular proliferation through controlling apoptosis [9, 13, 15, 42]. The MIR21 gene is transcribed via RNA polymerase II and is located in intronic regions of the transmembrane 49 gene (TMEM49; also referred to as VMP1) [10, 17]. The mature MIR21 sequence was first identified in human HeLa cells [22] and has since been predicted and verified to be present in the transcriptome of several other species including the pig. The anti-apoptotic capabilities of MIR21 in cancer cells are manifested through the ability to suppress critical apoptotic genes including programmed cell death 4 (PDCD4, previously referred to as neoplastic transformation inhibitor) [3, 16, 24, 33]. MIR21 interacts with *PDCD4* through binding with complementary sequence in the 3′UTR of *PDCD4* mRNA resulting in reduced translation and subsequently reduced protein abundance in oncogenic cell lines [3, 24]. Importantly the 3′UTR of pig *PDCD4* possesses a conserved MIR21 recognition sequence, particularly in the seed sequence. This suggests that if both MIR21 and *PDCD4* are present in the oocyte, MIR21 could impact PDCD4 protein abundance as the necessary accessory proteins for miRNA function are present in the oocyte during GVBD and progression to MII arrest.

Our working hypothesis that increased MIR21 abundance in the maturing cumulus oocyte complex of the pig is associated with posttranscriptional regulation of *PDCD4* expression in the oocyte and that suppression of MIR21 function during oocyte maturation will compromise subsequent embryonic development. The objective of this study was to determine expression patterns of MIR21 and demonstrate its potential interactions with PDCD4 in the cumulus oocyte complex (COC) during oocyte maturation in the pig. Here we demonstrate PDCD4 protein down regulation is temporally associated with MIR21 abundance increase during in vitro oocyte maturation. These data indicate a reduced ability of MIR21 to suppress PDCD4 protein abundance in the presence of a MIR21 inhibitor suggesting a biological interaction between MIR21 and PDCD4 mRNA occurs during in vitro oocyte maturation in the pig.

## Methods

Animal use was in accordance with the Guiding Principles for Care and Animals and procedures were approved by the Iowa State Institutional Animal Care and Use Committee.

### In vitro maturation

All chemicals were purchased from Sigma Chemical Co. (St. Louis MO) unless otherwise stated. Sow ovaries were obtained from a local abattoir for isolation of cumulus oocyte complexes (COCs) to be subjected to in vitro maturation (IVM) as previously described [41, 44]. Briefly, follicles (3–5 mm) were aspirated and COC were collected and washed in TL-Hepes with 0.1 % polyvinyl alcohol (PVA). Cumulus oocyte complexes were cultured in maturation media (Tissue Culture Media 199 (TCM-199)) containing 0.57 mM L-cysteine, follicle stimulating hormone (0.5 μg/mL), luteinizing hormone (0.5 μg/mL), and epidermal growth factor (10 ng/mL) for 42–44 h at 39.0 °C in 5 % CO_2_. Prior to in vitro maturation, an aliquot of GV stage oocytes for each replication were randomly selected from the COC pool. GV stage oocytes used for analysis were stripped of cumulus cells via vortex (6 to 8 min) in 1 % hyaluronidase in TL-Hepes-PVA. Following in vitro maturation oocytes were stripped of cumulus cells by vortexing 4–6 min in TL-Hepes-PVA supplemented with 1 % hyaluronidase, and Metaphase II arrested (MII) oocytes were identified by the presence of an extruded polar body. Cumulus cells before and after maturation and GV and MII oocytes (25 oocytes per pool) were snap frozen in liquid nitrogen and stored at −80 °C until used for quantitative reverse transcription PCR (RT-qPCR). Pools of GV and MII arrested oocytes from the same replications (50 oocytes per pool) were utilized for Western blot analysis.

### MIR21 expression in oocytes with and without LH and FSH during in vitro maturation

To determine the effect of LH and FSH on MIR21 expression in oocytes during in vitro maturation COCs were matured in defined maturation media with LH and FSH as described above or containing only LH, only FSH, or lacking both. COCs were washed four times and cultured in the designated hormone treatment in groups of 80–90 COCs per well. COCs were cultured for 42 h, denuded of cumulus cells by vortexing as described above and MII oocytes were identified by the presence of a polar body. This experiment consisted of four biological replications. Maturation rates, as defined by the percentage of oocytes achieving MII arrest, were recorded and MII oocytes from each treatment and replication were collected in pools and used for MIR21 expression analysis as described above.

### MIR21 expression in oocytes cultured with and without cumulus cells

To determine the effect of cumulus cell presence on MIR21 expression in oocytes during in vitro maturation we subjected COCs to one of three treatments: 1) standard in vitro maturation as described above using intact COCs, 2) in vitro maturation following cumulus cell removal and then utilization of detached cumulus cells for culture with denuded oocytes or, 3) denuded oocytes matured without the presence of cumulus cells. Cumulus cells were removed by gentle vortex in 1 % hyaluronidase in TL-Hepes, washed twice in 200 μL of maturation media and then resuspended in 200 μL of maturation media and added to the in vitro maturation culture plates which contained 300 μL maturation media and the denuded oocytes. Final volume of culture media for all plates was 500 μL and each well contained 75–85 oocytes. This experiment consisted of four biological replications. Maturation rates, as defined by the percentage of oocytes achieving MII arrest, were recorded and MII oocytes from each treatment and replication were collected in pools and used for MIR21 expression analysis as described above.

### MIR21 inhibition during in vitro maturation

Peptide nucleic acids (PNA) are artificially constructed oligonucleotides with strong affinity and specificity to endogenous nucleotides while resistant to nucleases making them ideal for miRNA inhibition [31]. We used an anti-MIR21 PNA (Panagene Inc. Daejeon, Korea) designed to specifically bind to and prevent MIR21 activity. A scrambled PNA with no predicted targets was used as a negative control (Panagene Inc., Daejeon, Korea). PNA oligonucleotides were diluted in maturation media at a stock concentration of 100 nM/μL and then added to maturation media on the day of COC collection to acquire a final concentration of 2.0 nM and 0.2 nM. A control group without PNA was used to evaluate the potential toxicity of the PNA.

Parthenogenetic activation of MII oocytes, used to remove the confounding effects of miRNA introduced by sperm, was performed with 50 oocytes from each treatment to determine developmental competence of embryos up to 60 h. MII oocytes were washed in a high calcium activation media (Mannitol 0.28 M, CaCl_2_ 1.0 mM, MgCl_2_ 0.1 mM, HEPES 0.5 mM and BSA 1 mg/mL), then placed between two electrodes covered with activation media and activated by two consecutive 30 μsec pulses at 1.2 kV/cm. Following activation, zygotes were washed and cultured in porcine zygote medium-3 (PZM3) at 39 °C in 5 % CO_2_ [21]. At 60 h embryos were evaluated for development and the number of embryos with four or more uniform blastomeres was recorded.

### Quantitative RT-PCR of oocytes and cumulus cells

Oocytes were collected and denuded of cumulus cells as described above. Oocytes from each stage of development and treatment were collected in pools of exactly 25 oocytes in 5 μL of PBS. As before using a precise number of oocytes per reaction we were able to avoid the introduction of additional variation associated with reference genes [41]. Both *PDCD4* and MIR21 analysis were analyzed from the same oocyte sample lysis. TaqMan™ Gene Expression Cells-to-Ct™ Kit (Applied Biosystems, Carlsbad, CA) was used to lyse oocytes and prepare samples for RT-qPCR. Lysis solution and DNase from the Cells-to-Ct kit were added to each oocyte pool at 4.95 and 0.05 μL, respectively, and incubated at RT for 5 min. Stop solution (0.5 μL) was added, incubated for an additional 2 min and placed on ice. Two μl of the sample lysis was added to each RT-qPCR reaction. *PDCD4* forward (5′-ACAGTTGGTGGGCCAGTTTATTGC-3′) and reverse (5′-CTTTGCGCCTTCCACCTTTAGACA- 3′) primers were used to determine mRNA abundance of *PDCD4* within each pool. QuantiTect® SYBR® Green RT-PCR Kit (Qiagen) was used for the RT-qPCR reaction for *PDCD4* according to manufacturer’s recommendations. The standard cycling conditions were 50 °C for 30 min, 95 °C for 15 min followed by 45 cycles of 95 °C for 15 s, 60 °C for 30 s, and 72 °C for 30 s followed by melting curve analysis.

MIR21 was quantified using TaqMan® MicroRNA Reverse Transcription kit (Applied Biosystems Carlsbad, CA) for the reverse transcription (RT) reaction and the primers and probe used were TaqMan® MicroRNA Assay for hsa-MIR21 (Applied Biosystems, Carlsbad, CA) according to manufacturer’s recommendations. The RT reaction was 20 μL consisting of 13 μL master mix, 3 μL primers, and 4 μL sample lysis. Reverse transcription conditions were 16 °C for 30 min, 42 °C for 30 min and 85 ° C for 5 min. The final volume for all RT-qPCR reactions was 20 μL which include 1.33 μL of the RT product, 1 μL TaqMan MicroRNA Assay (20x), 10 μL TaqMan 2X Universal PCR Master Mix and 7.67 nuclease free water. The thermal cycling conditions for the TaqMan MicroRNA RT-qPCR were 95 °C for 10 min, followed by 45 cycles of 95 °C for 15 s and 60 °C for 60 s. Fluorescent data acquisition was during the 60 °C extension step.

For analysis of MIR21 expression in cumulus cells total RNA was extracted from cumulus cells of GV stage and MII arrested oocytes using the mirVana RNA isolation kit (Life Technologies, Grand Island, NY), 10 ng of total RNA was utilized for the RT reaction conducted the same way as the oocyte samples. For cumulus cells, cycle threshold values were normalized to expression of another small RNA, RNU43, prior to comparison between stages and statistical analysis. All samples were assayed in duplicate. The comparative C_T_ method was used to calculate relative fold changes between samples as previously described [35].

### PDCD4 Western blot analysis

Pools of 50 denuded GV and MII oocytes within a replication were collected, washed in PBS, and stored at −80 °C until used for Western blot analysis. Oocyte pools were lysed in 2.5 μL of 5X SDS (total sample volume 12.5 μL) at 95 °C for 4 min followed by 1 min on ice and then centrifugation at 1000 rpm for 1 min at RT. Samples were then loaded into a 4–20 % Tris glycine gel (Lonza PAGEr® Gold Precast Gels). The BioRad Mini PROTEAN Tetra System was used to run the gel at 60 V for 30 min followed by 120 V for 90 min. The gel was transferred to a nitrocellulose membrane for 1 h at 100 V at 4 °C. In addition to utilizing an exact number of oocytes per lane, Ponceau S staining was used to confirm relative transfer efficiency between lanes and equivalent total protein loading per lane. Membrane blocking was conducted using 5 % milk in PBST (PBS with 0.5 % Tween 20) for 1 h at RT. A rabbit anti-PDCD4 monoclonal antibody (Abcam, ab79405) was added (1:1000 dilution) to the membrane in 0.5 % milk in PBST overnight at 4 °C and a negative control membrane lacking primary antibody was also conducted. Following primary antibody incubation, the membranes were washed with PBST three times at RT for 10 min each. Donkey anti-Rabbit IgG (Amersham™ ECL™ NA934) was incubated (1:2000) with the membrane for 1 h at RT. The membrane was then washed three times for 10 min each at RT. Horseradish peroxidase substrate (Millipore, Billerica, MA) was added to the membrane for 1 min in the dark. The membrane was then exposed to x-ray film and developed for visualization. Average pixel intensity for the protein corresponding to 52 kDa (PDCD4 molecular weight) was conducted using Image J [1].

### MIR21 in situ hybridization in the developing follicle

Ovaries were preserved in 4 % paraformaldehyde and utilized for *in situ* hybridization to determine MIR21 expression. Ovary sections (5 μm) were then mounted on slides for analysis. Each section was subjected to CitriSolv (Fisher Scientific) twice for five minutes rehydrated in two changes each of 100 % ethanol for 3 min, followed by 95 % ethanol for one minute and finally 80 % ethanol for one minute. Slides were immersed in heated citrate buffer (95 °C) for 30 min and then cooled to RT. Once at RT slides were blocked for 30 min with 5 % BSA. Slides were then placed in hybridization solution for 1 h at 65 °C. The 5′ fluorescein labeled miRCURY LNA detection probe (Exiqon) was added and slides were incubated in high humidity overnight at 65 °C. Slides were then washed in saline-sodium citrate (SSC) solution and PBST at RT. Anti-fade DAPI was added and a cover slip was placed over each section. Primary and secondary follicles were imaged at 400X and tertiary follicles were imaged at 200X with a Leica microscope.

### Statistical analysis

PROC MIXED of the Statistical Analysis System was used to determine statistical differences of all data including percentage maturation and differences in C_T_ value for RT-qPCR data. Significance (*P* < 0.05) was determined for the model and least-square means was used to determine significant differences between samples. The effect of oocyte stage on MIR21 and PDCD4 expression (C_T_ value) was determined. Treatment effect for the PNA inhibitor of MIR21, presence or absence of gonadotropins, and the presence or absence of cumulus cells during in vitro maturation on MIR21 expression was evaluated. Replication was included as a covariate. Graphs depicting percent change were adjusted to reflect the GV stage as 100 % and all other treatments are relative to GV. Data are displayed as mean ± SEM.

## Results

### MIR21 expression is temporally regulated during porcine cumulus oocyte complex maturation

**Fig. 1:**
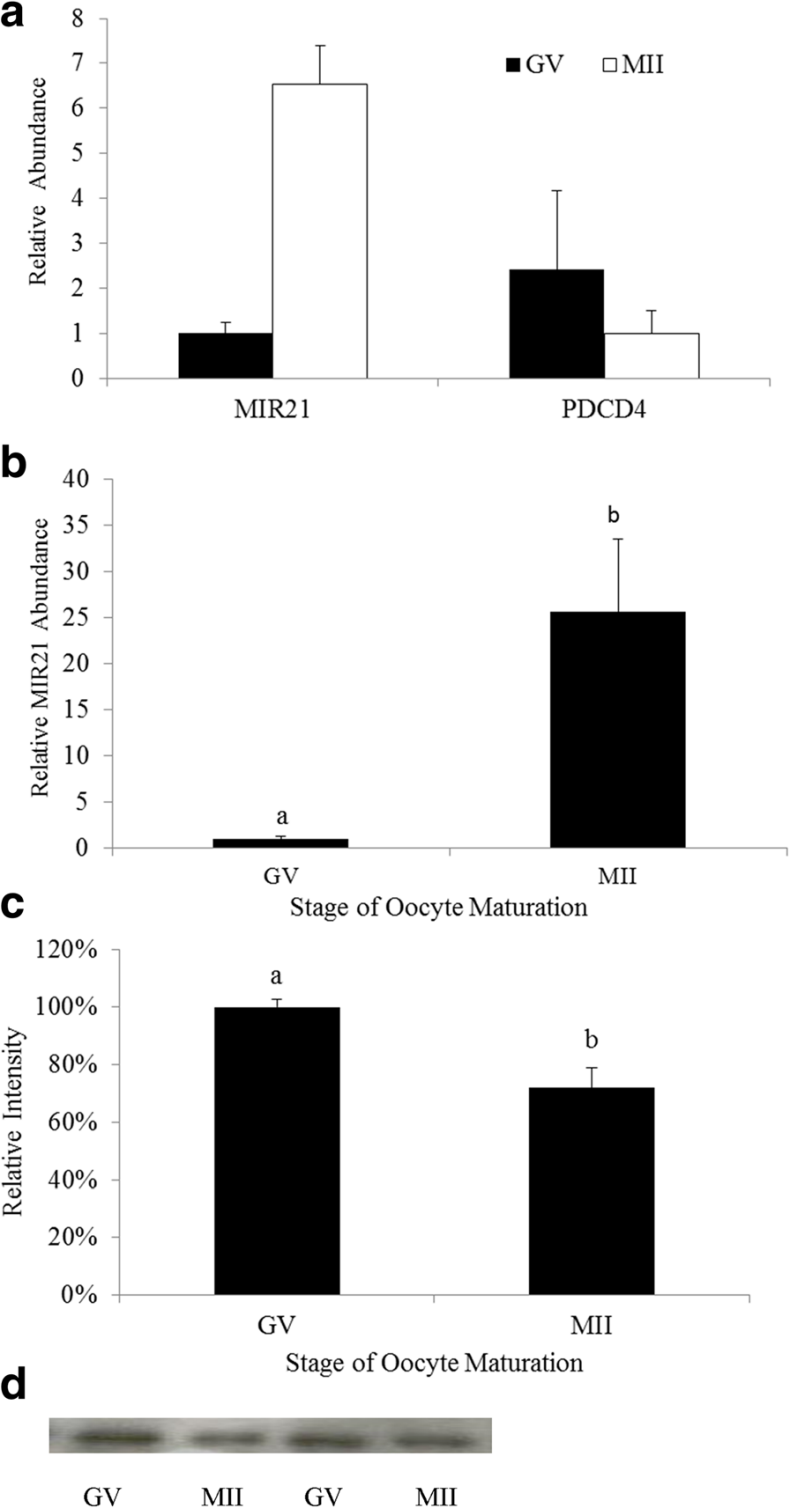
MIR21 expression is significantly increased in cumulus cells and oocytes during in vitro maturation. **a** RT-qPCR analysis for MIR21 and *PDCD4* mRNA in GV stage and MII arrested oocytes (*n* = 4). **b** RT-qPCR analysis for MIR21 in cumulus cells isolated from GV stage and in vitro matured oocytes (*n* = 4). **c** PDCD4 Western blot analysis of GV stage and MII arrested oocytes. Pixel intensity was quantified with ImageJ and demonstrates a decrease in PDCD4 protein abundance in MII oocytes compared with GV oocytes (*n* = 3). **d** Representative Western blot of PDCD4 protein detection in oocytes representing the 52 KDa band. ^a,b^Means ± SEM with different superscripts are different (*P* < 0.05)

To determine the relationship between MIR21 and its putative target *PDCD4*, during in vitro maturation, RT-qPCR and Western blot analysis were utilized to evaluate expression of MIR21 and PDCD4 in GV and MII stage oocytes and cumulus cells. During the transition from GV to MII, MIR21 was up-regulated approximately 6-fold in oocytes (*P* = 0.001, Fig. 1a) and approximately 25-fold in cumulus cells (*P* = 0.003, Fig. 1b). In the same samples *PDCD4* mRNA abundance was not statistically different (*P* = 0.34, Fig. 1a). Western blot analysis demonstrated PDCD4 protein abundance was reduced (*P* = 0.02) in oocytes during the transition from GV stage to MII arrested oocytes (Fig. 1c and d).

### Gonadotropins in maturation media influence maturation rate but not MIR21 abundance in MII arrested oocytes

**Fig. 2:**
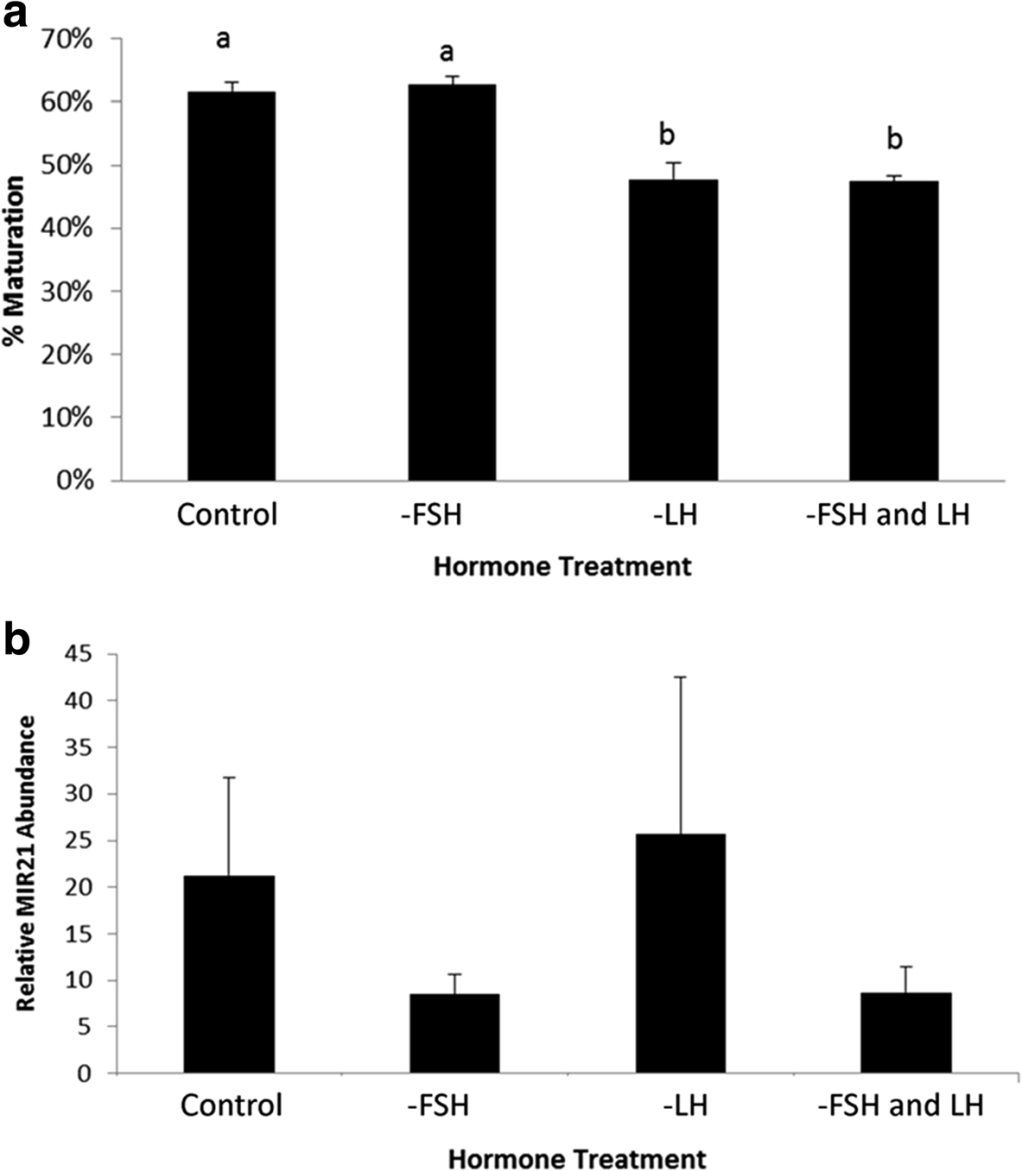
Luteinizing hormone and follicle stimulating hormone affect oocyte maturation to MII arrest and the lack of FSH numerically decreased MIR21 expression in MII oocytes. **a** Maturation rates for oocytes cultured with both LH and FSH (Control), without FSH (− FSH), without LH (−LH) and without LH and FSH (−LH and FSH). **b** MIR21 relative expression for GV oocytes and MII oocytes cultured with LH and FSH, without FSH, without LH and without LH and FSH. ^a,b^Means ± SEM with different superscripts are different (*P* < 0.05)

To determine the effect of gonadotropins on MIR21 expression during in vitro oocyte maturation, we matured COCs with and without luteinizing hormone (LH) and follicle stimulating hormone (FSH). Maturation rates were not different between media containing only LH (62.7 ± 1.4 %) compared with control maturation (61.7 ± 1.2 %, Fig. 2a). However, progression to MII arrest was significantly decreased (*P* < 0.001) when COCs were cultured in maturation media with only FSH (47.8 ± 2.5 %) or lacking both FSH and LH (47.4 ± 0.9, Fig. 2a). MIR21 expression in MII arrested oocytes was not significantly different between the control and treatment groups (*P* = 0.68) (Fig. 2b).

### Cumulus cells influence oocyte maturation but not MIR21 abundance in MII arrested oocytes

**Fig. 3:**
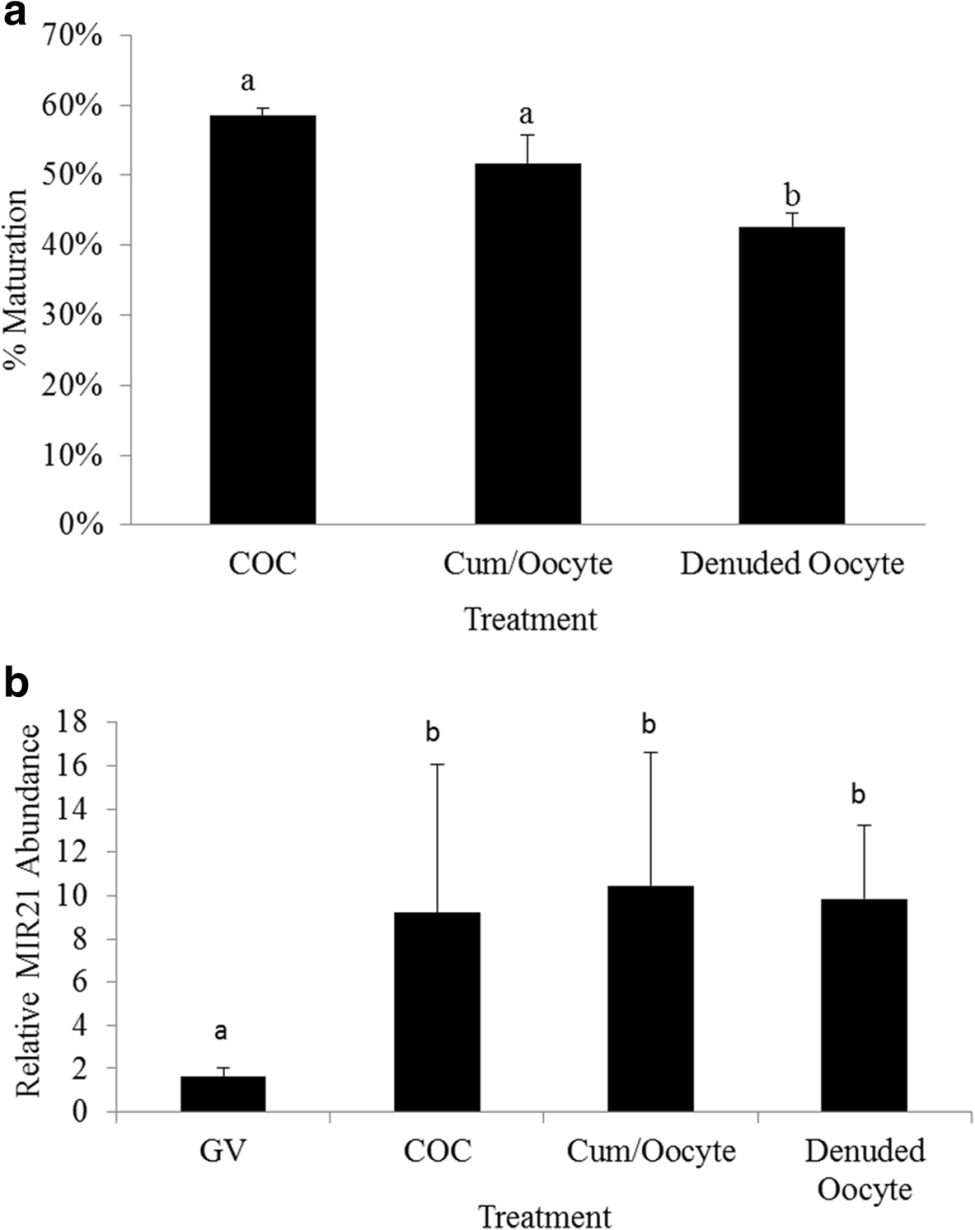
Cumulus cells influence oocyte maturation but not MIR21abundance in MII arrested oocytes. **a** Maturation rates for intact COC, denuded oocytes cultured with cumulus cells, and denuded oocytes. **b** Expression of MIR21 in GV oocytes, and MII arrested oocytes from intact COC, denuded oocytes cultured with cumulus cells, and denuded oocytes. ^a,b^Means ± SEM with different superscripts are different (*P* < 0.05)

To determine the impact of cumulus cell presence on MIR21 abundance in the oocyte during in vitro maturation we compared MIR21 expression in MII arrested oocytes following maturation of intact COCs, denuded oocytes cultured in the presence of cumulus cells and denuded oocytes cultured without cumulus cells. Maturation rate for control COCs was 58.4 ± 1.5 % which tended to be greater than denuded oocytes cultured with cumulus cells (51.2 ± 4.0 %, *P* = 0.07). However, denuded oocytes cultured without cumulus cells had significantly lower (42.5 ± 2.0 %) maturation rates than intact COCs (*P < 0.05*) and denuded oocytes cultured with cumulus cells (*P* < 0.05, Fig. 3a). MIR21 expression in MII arrested oocytes was not affected by the presence or absence of cumulus cells during maturation (*P* = 0.82). GV oocytes had lower MIR21 expression (*P* < 0.05) compared to MII arrested oocytes for all treatments (Fig. 3b), consistent with data from the experiment presented in Fig. 1.

### Inhibition of MIR21 affects oocyte maturation and PDCD4 protein expression

Using a fluorescently labeled MIR21 antagonist we first demonstrated its ability to translocate into both the cumulus cell and the oocytes (data not shown). All oocytes exposed to the fluorescently labelled inhibitor demonstrated a measurable level of fluorescence compared with oocytes treated with a non-fluorescently tagged control inhibitor, although some variability in the intensity of the fluorescence existed between oocytes. Anti-MIR21 antisense oligonucleotides were added to maturation media during oocyte maturation to determine the effects of MIR21 inhibition on maturation rate and PDCD4 expression in MII arrested oocytes. Inhibition of MIR21 using an anti-MIR21 PNA during oocyte maturation decreased (*P* < 0.01) the percentage of oocytes achieving MII after 42 h of culture (Table 1). Oocytes cultured in the presence of the negative control inhibitor (NC-PNA 2.0 nM) had similar maturation rates compared to control maturation conditions (*P* > 0.05) and greater (*P* < 0.05) maturation rates compared to the MIR21 inhibited oocytes (Table 1).

**Table 1:** Oocyte development to MII arrest and 4-cell stage of embryonic development at 60 h following parthenogenetic activation

Treatment group^c^	Total oocytes matured^d^	Percentage MII arrested oocytes^e^	Percentage 4-cell or greater at 60 h^f^	Average blastomere # at 60 h^g^
Control	1646	55.4 ± 3.6^a^	73.0 ± 5.7^a^	5.8 ± 1.2 (*n* = 16)
NC-PNA	1136	49.0 ± 2.5^a^	60.2 ± 15.8^ab^	6.3 ± 1.4 (*n* = 12)
Anti-MIR21 PNA	1639	33.7 ± 3.6^b^	41.7 ± 12.1^b^	4.8 ± 1.4 (*n* = 12)

^a,b^Values with different superscripts in the same column are significantly different (*P* < 0.05)

^c^Treatments include: control maturation conditions, NC-PNA (2.0 nM), anti-MIR21 PNA (2.0 nM)

^d^Total number of GV oocytes matured for each treatment from five replications

^e^Percentage of MII arrested oocytes from each treatment. Mean ± SEM

^f^Percentage of embryos achieving 4-stage or greater within 60 h following parthenogenetic activation of MII arrested oocytes. Mean ± SEM

^g^Average number of blastomeres at 60 h based on a subset of embryos stained with nuclear stain DAPI

**Fig. 4:**
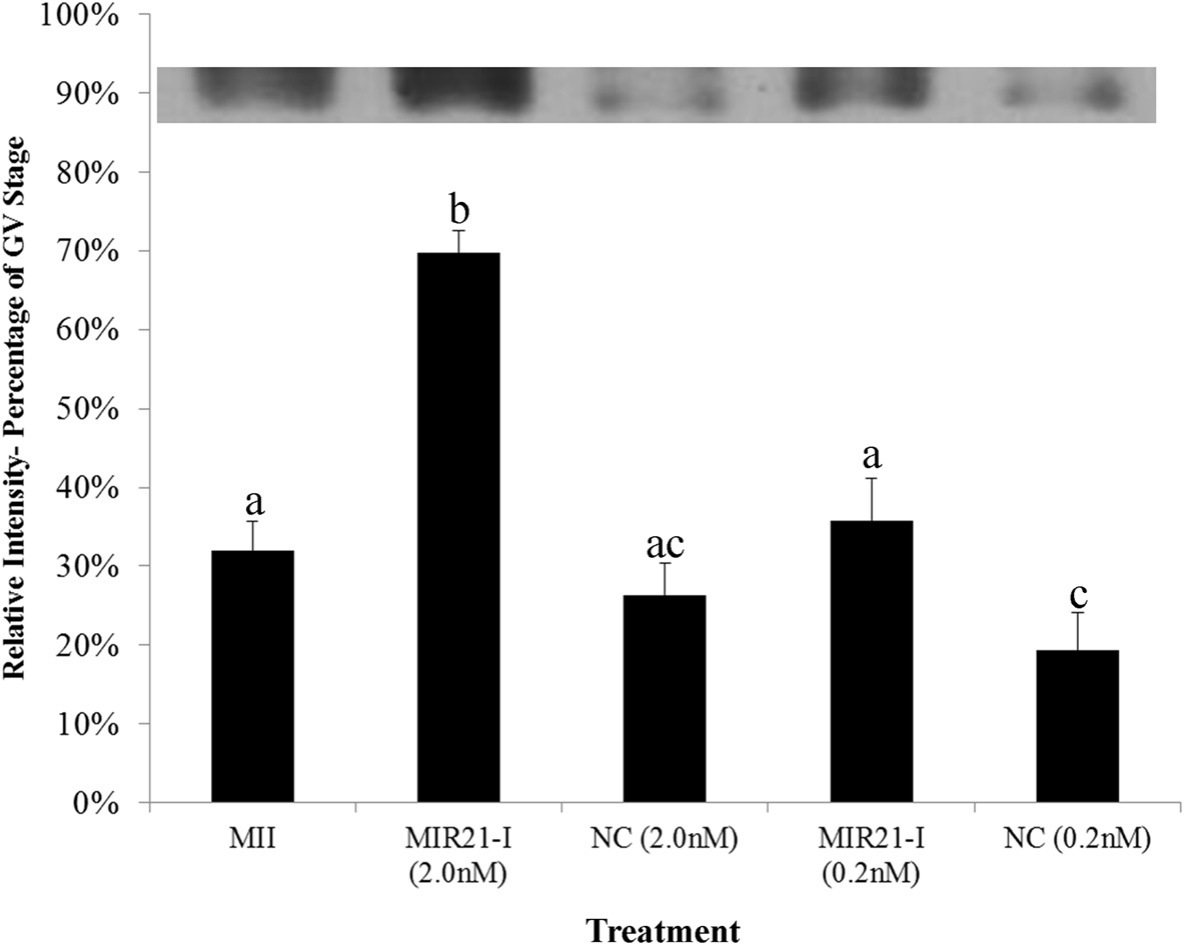
PDCD4 protein expression following MIR21 inhibition during IVM. PDCD4 expression abundance is presented relative to PDCD4 expression in GV oocyte samples (*n* = 4). From left to right: Control MII arrested oocytes, MII arrested oocytes in vitro matured in the presence of high concentration (2.0 nM) of the anti-MIR21 PNA or negative control PNA, and MII arrested oocytes in vitro matured in the presence of low concentration (0.2 nM) of the anit-MIR21 PNA or negative control PNA. Data are presented following normalization to the mean GV stage oocyte band intensity. ^a,b^Means ± SEM with different superscripts are different (*P* < 0.05). Inset is a representative Western blot of PDCD4 protein expression

The effect of MIR21 inhibition on PDCD4 protein abundance was analyzed by Western blot with pools of 50 oocytes within each treatment and presented as a percentage of GV stage PDCD4 abundance. Western blot analysis demonstrated significantly lower PDCD4 abundance in control MII arrested oocytes (31.9 % of GV) compared to MII arrested oocytes matured in the presence of 2.0 nM MIR21 antagonist (69.7 % of GV; *P* < 0.05). PDCD4 abundance in MII oocytes subjected to IVM in the presence of low (0.2 nM) MIR21 inhibitor concentration was similar (26.4 % of GV stage) to control MII oocytes and oocytes cultured with either concentration of NC-PNA possessed a similar quantity or less of PDCD4 compared to GV oocytes (Fig. 4).

### MIR21 inhibition during in vitro maturation impacts parthenogenetic embryo development

Metaphase II arrested oocytes were parthenogenetically activated (to eliminate confounding impact of sperm-borne miRNA) following in vitro maturation to test the hypothesis that MIR21 inhibition during in vitro oocyte maturation negatively impacts early embryo development prior to activation of the embryonic genome at the 4-cell stage of development. The number of embryos achieving the 4-cell stage of development or greater within 60 h post activation was significantly (*P <* 0.05) affected by MIR21 inhibition during in vitro maturation (Table 1). Development to the 4-cell stage or greater within 60 h post activation was greatest for control oocytes (73.0 ± 5.7) compared with oocytes matured in the presence of a MIR21 inhibitor (41.7 ± 12.1) or in the presence of a negative control PNA (60.2 ± 15.8).

### MIR21 abundance in the developing pig follicle

**Fig. 5:**
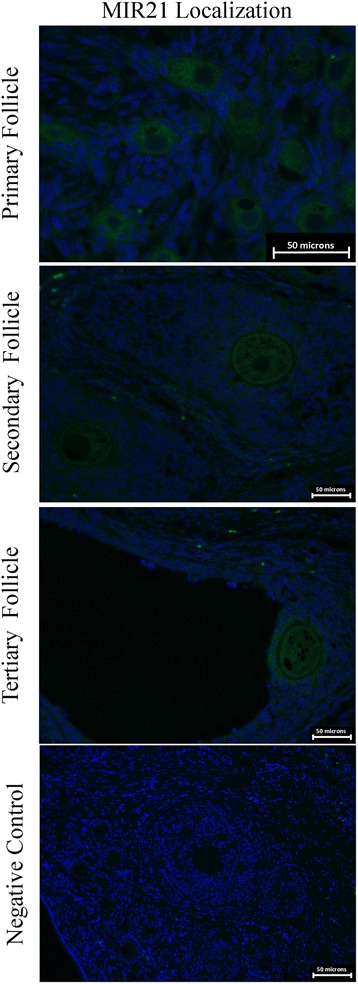
*In situ* hybridization of MIR21 in gilt ovaries to identify MIR21 expression during follicle development. Scale bars represent 50 micron in all images. Images are representative of eight biological replications. MIR21 expression was designated green and nuclei (DAPI staining) designated blue. Laser intensity and exposure time was consistent for all images captured

*In situ* hybridization of MIR21 demonstrated expression throughout the granulosa cells and the oocyte (Fig. 5). In primary follicles expression is present in the oocyte with faint expression in the surrounding cells. However secondary and tertiary follicles express MIR21 more abundantly in the oocyte as well as the surrounding cumulus cells compared to primary follicles. Based on staining intensity, MIR21 abundance appears greatest in oocytes and granulosa cells of tertiary follicles.

## Discussion

While abundant in the developing oocyte and mouse ovary [11, 14] miRNA expression and function during pig oocyte maturation and early embryo development is only in the initial stages of characterization [18, 41]. The potential ability of MIR21 to interact with *PDCD4* leading to posttranscriptional gene regulation of PDCD4 protein expression in the maturing pig oocyte as demonstrated herein has also been described in several types of cancer cells [3, 15, 16, 29, 33, 42]. The interaction between MIR21 and PDCD4 is likely conserved in pigs as the MIR21 target recognition sequence in the 3′UTR of human and pig PDCD4 is 97 % similar with 100 % similarity in nucleotides responsible for recognition by the MIR21 seed sequence.

Although a multitude of reports demonstrating the biological activity of miRNA in somatic cells exists miRNA function in maturing oocytes has been debated, primarily the result of the conditional knock-out during oocyte maturation of enzymes needed for both canonical and non-canonical miRNA biogenesis pathways. Loss of DICER function, essential for both siRNA and canonical miRNA, in developing oocytes results in the production of non-viable oocytes [38]. Alternatively, using the same ZP3-cre conditional knock-out approach with a floxed DGCR8 allele, mice lacking the ability to produce miRNA through the canonical biogenic pathway still produced viable oocytes, despite reduced fecundity [25, 36]. This suggests that while miRNA activity in mature mice oocytes may be suppressed, miRNA may still contribute to the developmental competency of the subsequently produced embryo. This study tested the hypothesis that increased MIR21 abundance in the maturing cumulus oocyte complex of the pig is associated with posttranscriptional regulation of *PDCD4* expression in the oocyte and that suppression of MIR21 function during oocyte maturation would compromise subsequent embryonic development.

Utilizing miRNA microarray and small RNA sequencing, the expression of numerous miRNA in the maturing porcine cumulus oocyte complex have been previously demonstrated [41]. The current study further characterizes the temporal relationship between increased mature MIR21 abundance and decreased PDCD4 protein abundance during in vitro maturation of pig oocytes. In cumulus cells, MIR21 abundance increases approximately 25-fold during in vitro maturation. This is consistent with reports in humans [4] and in mice demonstrating increased MIR21 expression in granulosa cells in response to LH [11]. Because MIR21 abundance changes appear to occur rapidly during in vitro maturation, it is of interest if MIR21 expression in the pig cumulus oocyte complex is also responsive to the gonadotropins. To examine this, the ability of both LH and FSH to affect MIR21 expression during in vitro maturation was examined. While exclusion of LH from maturation media negatively impacted oocyte maturation, as demonstrated by others [45], a significant effect of the gonadotropins on MIR21 abundance during in vitro maturation was not detected.

Because changes in MIR21 abundance in the oocyte did not appear to be greatly influenced by LH and FSH other mechanisms that may contribute to changes in MIR21 abundance were also investigated. A potential mechanism explaining the observed increase of MIR21 abundance in IVM derived MII arrested oocytes compared to GV oocytes is that MIR21 could be transported into the oocyte from the cumulus cells during maturation. This is plausible as other factors have been demonstrated to be translocated between the maturing oocyte and the surrounding cumulus oophorus [19]. Oocyte - cumulus cell communication is bi-directional and required for normal cumulus cell gene expression and oocyte maturation [20] and gap junctions allow the transit of molecules less than 1000 Da including ATP, sodium, chloride, calcium ion and cAMP [2, 26]. Therefore it is feasible that miRNA could also be transported into the oocyte from surrounding cumulus cells. In addition to gap junctions, small molecule transport between cells has also been demonstrated to occur through exosomes and other microvesicles that can be secreted and taken up by neighboring cells [39, 40]. The potential for these mechanisms to contribute to MIR21 abundance increasing in the oocyte was examined by culturing denuded oocytes during IVM in the presence or absence of cumulus cells. While a reduction in maturation rate was observed as has been previously demonstrated [43], MIR21 abundance in the oocytes that did achieve MII arrest was not affected by the presence of cumulus cells suggesting the increased MIR21 observed is at least in part, the result of oocyte specific mechanisms.

Taken together neither gonadotropins nor the presence of cumulus cells during IVM is demonstrably responsible for increased MIR21 abundance consistently observed in MII arrested oocytes compared to their GV stage counterparts. It has also been observed that GVBD does not occur in the majority of porcine oocytes until approximately 16–24 h of in vitro maturation [30]. Therefore, it remains possible that the increased abundance of MIR21 in the MII arrested oocyte occurs as a transcriptional response in the oocyte during the initial phases of in vitro maturation prior to GVBD in the oocyte.

These findings taken together suggest the potential for MIR21 and miRNA in general, to impact protein expression in the maturing oocyte having implications regarding the developmental ability of subsequently produced embryos. Oocyte growth and development begins prior to antral follicle development and it is unclear when activation of MIR21 expression occurs or what mechanism is primarily responsible for this observation. The cumulus oocyte complexes collected for this study were from 3 to 5 mm antral follicles, prior to GVBD, and may be capable of transcribing primary MIR21 (pri-MIR21) transcript that can be further processed into mature MIR21 during maturation. Future studies will include an analysis of pri-MIR21 abundance prior to and during oocyte maturation which may yield insight into the mechanisms contributing to the current observations.

Several promoters have been identified upstream to the pri-MIR21 transcription start site containing predicted consensus sequences for binding of AP-1 and signal transducer and activator of transcription 3 (STAT3) [17]. In certain cancers, the interaction between MIR21 and PDCD4 is necessary for maximal AP-1 activation. AP-1 activation is suppressed by PDCD4, however, AP-1 induction of MIR21 and subsequent posttranscriptional regulation of PDCD4 allows further and more sustained AP-1 activation [17, 37]. In addition to AP-1, STAT3 is another transcription factor that has also been documented to induce pri-MIR21 transcription [23]. It is possible that STAT3 could be related to the expression of MIR21 during oocyte maturation and early embryo development as leptin (an adipokine) has been demonstrated to increase STAT3 expression during bovine embryo culture and is associated with reduced apoptosis in bovine blastocysts [7]. In addition, leptin has been shown to increase STAT3 expression in both cumulus cells and oocytes during in vitro maturation and enhance the oocytes ability to complete meiosis [32].

## Conclusion

In summary, these data demonstrate MIR21 miRNA and PDCD4 protein have a reciprocally inverse, temporal expression pattern during oocyte maturation, and that inhibition of MIR21 results in an increased abundance of PDCD4 protein. MIR21 expression in MII arrested oocytes matured in vitro was not affected by gonadotropins or the presence of cumulus cells during in vitro maturation. It is possible that increased MIR21 expression in the oocyte occurs earlier during oocyte recruitment or during initial in vitro maturation prior to GVBD and warrants further investigation. Obtaining an understanding of MIR21 and its mechanism is necessary to further understand molecular regulation of oocyte maturation and early embryo development in the pig. Understanding molecular mechanisms of early embryo development will provide a more comprehensive understanding of mammalian reproduction that can be fundamental in developing strategies to improve reproductive efficiency.

## Abbreviations

3′UTR: 3′ untranslated region
BSA: bovine serum albumin
COC: cumulus oocyte complex
GVBD: germinal vesicle breakdown
IVM: in vitro maturation
MII: metaphase II
MIR21: microRNA-21
miRNA: microRNA
mTBM medium: modified Tris-buffered medium
PBS: phosphate buffered saline
PBST: phosphate buffered saline containing 0.1 % Tween-20
PDCD4: programmed cell death 4
PNA: peptide nucleic acids
PTGR: posttranscriptional gene regulation
PVA: polyvinyl alcohol
PZM3: porcine zygote medium 3
RT: room temperature
TCM-199: tissue culture media 199
